# Complementary Design of Two Types of Signals for Avionic Phased-MIMO Weather Radar

**DOI:** 10.3390/s26020423

**Published:** 2026-01-09

**Authors:** Zhe Geng, Ling Wang, Fanwang Meng, Di Wu, Daiyin Zhu

**Affiliations:** 1 College of Electronics and Information Engineering, Nanjing University of Aeronautics and Astronautics, Nanjing 211106, China; tulip_wling@nuaa.edu.cn (L.W.); wudi82@nuaa.edu.cn (D.W.); zhudy@nuaa.edu.cn (D.Z.); 2 AVIC LEIHUA Electronic Technology Research Institute, Wuxi 214063, China; mengfw001@avic.com

**Keywords:** weather radar, waveform design, phased-MIMO radar, sidelobe control, beamforming

## Abstract

An avionic weather radar antenna should be able to operate in multiple modes to cope with the change in resolution and elevation coverage as an aircraft approaches a storm cell that could expand 10 km in elevation. To solve this problem, we propose the addition of four auxiliary antenna (AuxAnt) arrays based on the phased-MIMO antenna structure to the existing avionic weather radar for future field data collection missions. Two types of signals are employed: the Type I signal transmitted by AuxAnt 1 and 2 is designed based on a non-overlapping subarray configuration, with Subarray 1 and 2 dedicated to the transmission of long and short pulses, respectively, so that the near-range blind zone is mitigated. Leveraging the waveform design and beamforming flexibility provided by the phased-MIMO antenna, pulse compressions based on frequency modulation and phase-coding are employed for wide and narrow main beams, respectively. To suppress the range sidelobes, adaptive pulse compression is used at the receiver end in substitute of the conventional matched filter. In contrast, the Type II signal transmitted by AuxAnt 3 and 4 is designed based on the contextual information so that the transmitted beampatterns have specific sidelobe levels at certain directions for interference suppression. The advantages of the proposed signaling strategy are verified with a series of ingeniously devised experiments based on real weather data.

## 1. Introduction

The conventional weather radar antenna functional architecture sets strict constraints on waveform design, subarray segmentation, beamforming, as well as scan strategy. For example, the legacy avionic weather radar requires the pilot to adjust the antenna tilt angle manually. The most distinguishable advantage of the state-of-the-art airborne weather radar in service, Honeywell RDR4000, is that it uses up to 17 tilt angles and the Volumetric Buffer algorithms to directly measure all the weather cells ahead of the aircraft [1]. However, under the assumption that the information contained in each scan is of equal importance, Honeywell RDR4000 employs consecutive scans with one-degree increments, and each scan takes a similar time. In reality, the contributions made by beams pointing at different directions are never equal. For example, for ground-based weather radars, low-level elevations deserve more attention than higher altitudes. Therefore, the Supplemental Adaptive Intra-Volume Low-Level Scan (SAILS) strategy (2013) and the Mid-Volume Re-scan of Low-Level Elevations (MRLE) strategy (2018) employed by the NEXRAD allow the operator to re-scan a desired number of low-Level elevations [2]. Another effective strategy employed by NEXRAD for efficient data collection, the Automated Volume Scan Evaluation and Termination (AVSET) strategy, allows the beam to skip elevation angles that do not contain any precipitation echoes [3]. The operator may choose to invoke AVSET and either the SAILS or MRLE functions. SAILS and MRLE cannot be used together and the software will automatically toggle one off when the other is commanded on. AVSET will operate with both SAILS and MRLE [4]. This exemplifies the significance of updating the beam scanning strategy based on the contextual information.

Although there is plenty of time for the ground-based weather radar to re-scan the directions of interest in a sequential manner, simultaneous multibeam scanning is more suitable for an avionic weather radar mounted on an aircraft moving at a speed of 250 kts in descent. The adoption of phased-array radar (PAR) technology for weather applications is widely seen as the next step in the development of weather surveillance systems [5,6]. In addition to the conventional phased-array technique, hybrid antenna architectures such as the subarray-based multiple-input multiple-output (MIMO) radar also attract an increasing amount of attention. For example, the phased-MIMO radar architecture proposed by Hassanien et al. is a subarray-based hybrid of a phased-array radar and MIMO radar. The antenna elements within each subarray coherently transmit a waveform, while the waveforms transmitted from each subarray are orthogonal to each other [7]. Phased-MIMO radar could employ either non-overlapped or overlapped subarrays. Compared with coherent MIMO radar, phased-MIMO radars with overlapped subarrays offer higher SNR due to the coherent processing gain [8]. Moreover, they also support flexible signaling strategies [9,10,11,12].

The existing research on weather observation for PAR mainly concentrates on ground-based multifunctional radars operating at S-band. For example, the multifunction phased-array radar (MPAR) developed at the MIT Lincoln Laboratory is 4m in diameter and consists of 76 panels, with each panel composed of 8×8 antenna elements [6,13]. The MPAR is further segmented into 24 overlapped antenna subarrays, with each subarray containing 2×4 panels [13]. In 2018, a mobile weather radar based on the ten-panel demonstrator (TPD) made of ten MPAR panels arranged as a 2×5 matrix was developed [5]. The Horus radar developed by Palmer et al. has an aperture of 2 m and is made of 25 panels, with each panel consisting of 8×8 antenna elements, which lead to extreme flexibility in subarray segmentation and waveform design.

Although the existing weather radars equipped by transport and business aircrafts are mostly single-pol, supported by the rapid development of solid-state devices, dual-polarization and dual-band airborne weather radar designs have evolved from theoretical research to practical application [14,15,16]. In 2012, University of Oklahoma developed an airborne weather radar system called Polarimetric Airborne Radar Operating at X-band (PARADOX1), which employs adaptive pulse compression (APC) processing for range and antenna sidelobe suppression [17,18]. The APC technique on the receiving side was proposed by Blunt et al. in 2006 [19,20], and adaptively estimates which pulse compression filter to use for each range cell based on RMMSE and places nulls at range offsets corresponding to nearby large scatterers. This design later extended to PARADOX2, which was tested against NASA flight campaign data from 2015 [21]. In 2024, Li et al. presented an avionic dual-pol weather data simulation framework based on the invariant imbedding T-matrix (IITM) method, where the weather data collected by the KDGX ground-based radar were used as the ground truth [22]. In 2025, Chandrasekar et al. presented a simulation-based assessment of differential reflectivity under the assumption of a phased-array radar mounted on an airborne platform for both convective and stratiform precipitation events, where true and measured differential reflectivity were compared [23]. These works not only lay the foundation for developing correction strategies to improve the quality and reliability of airborne phased-array radar systems, but also reveal the importance of avionic dual-pol weather data collection.

**Figure 1:**
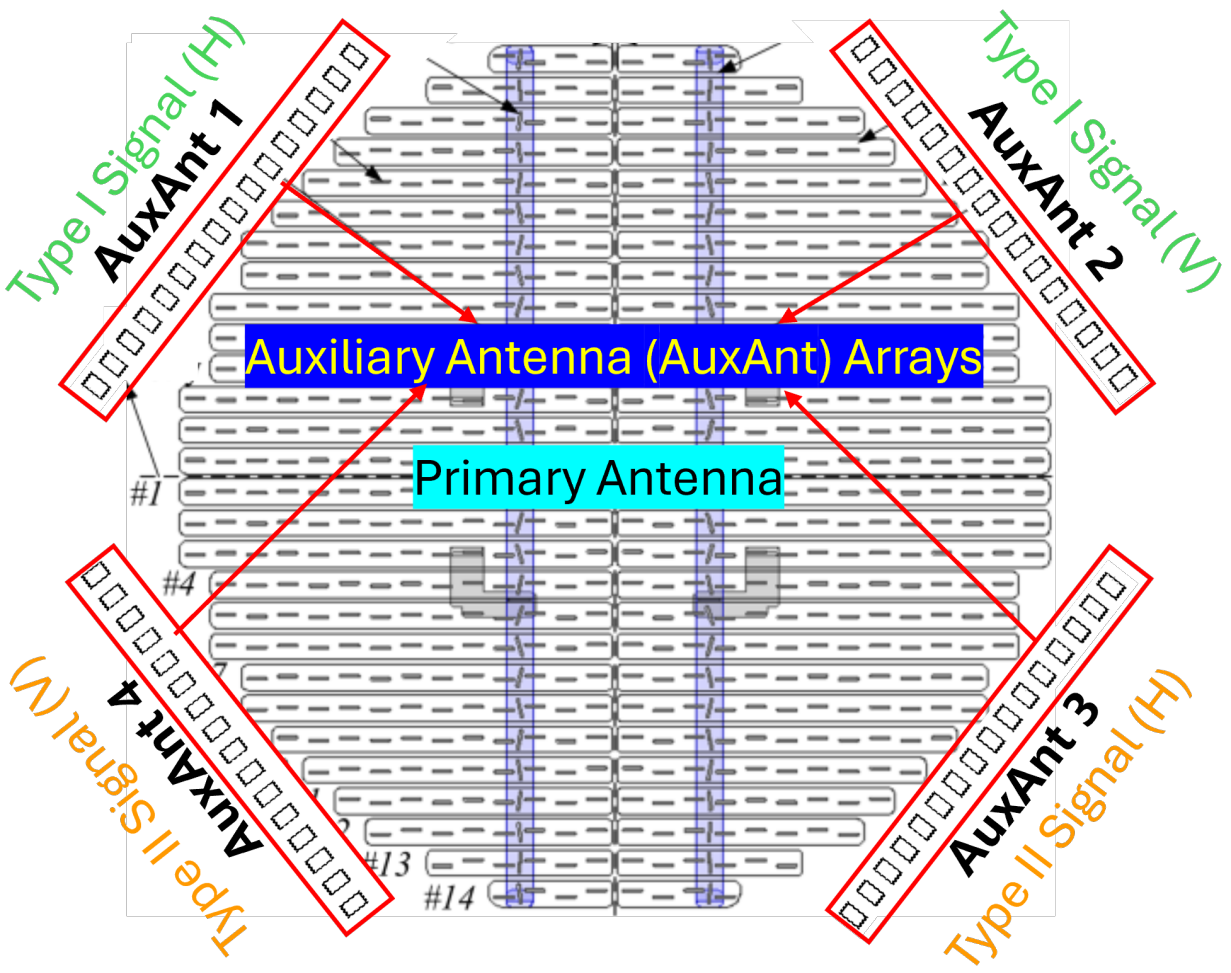
Four auxiliary antenna (AuxAnt) arrays are added to the existing avionic weather radar antenna. AuxAnt 1–4 are all based on the phased-MIMO technique, and provide extra waveform design and beamforming flexibility.

**Figure 2:**
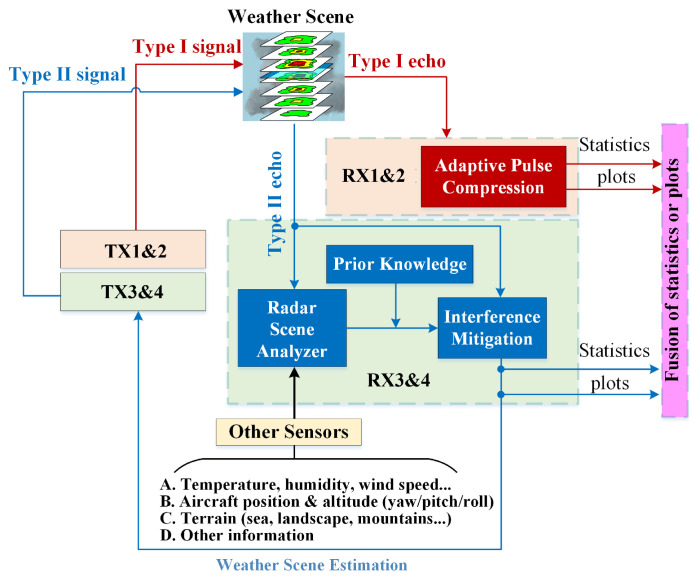
The overall flowchart of the proposed dual-channel meteorological data fusion scheme.

To facilitate the development of MPAR while meeting the design constraints for airborne weather radars in size, wight, power (SWaP), as well as cost, we propose adding auxiliary antenna arrays to the primary circular antenna array that is currently employed by the aircraft, which is illustrated in Figure 1. AuxAnt 1 transmits the horizontally polarized Type I signal, AuxAnt 2 transmits the vertically polarized Type I signal, AuxAnt 3 transmits the horizontally polarized Type II signal, and AuxAnt 4 transmits the vertically polarized Type II signal. To mitigate the near-range blind zone, AuxAnt 1 is divided into two non-overlapping subarrays to transmit the long pulse and the short fill pulse, respectively, which is equivalent to the twin transmit–receive channel architecture employed by PX1000 [24,25,26]. To minimize the range sidelobes, fast adaptive pulse compression (FAPC) based on the Reiterative Minimum Mean-Square Error (RMMSE) is employed as a substitute for the classic matched filter [17,18,19,20]. In contrast, the Type II signal transmitted by AuxAnt 3 and 4 is not designed in advance, but is adaptively generated. The overall flowchart of the proposed dual-channel meteorological data fusion scheme is illustrated in Figure 2.

The Type II signal transmitted by AuxAnt 3 and 4 is determined by the data collected by other sensors that are used as the input for the radar scene analyzer (RSA) and other prior knowledge so that the transmit beampatterns have specific sidelobe levels at certain directions for interference suppression. These data include (a) meteorological information, such as the temperature, humidity, and zonal/meridian/vertical wind speed; (b) aircraft kinemics, such as the position of the aircraft and the attitude (yaw/pitch/roll); (c) the terrain (i.e., sea, landscape, mountains, etc.), from which statistical properties are derived; and (d) other information, such as the data collected by the lightning sensor, turbulence probe, imaging sensor, and ground-based/satellite-based weather radars. By employing the proposed auxiliary antenna arrays, the beam scan strategy in the elevation domain becomes more flexible.

The major advantages of the proposed architecture include (i) an additional degree of freedom by forming multiple independent transmit–receive chains and dual-pol weather measurements; (ii) lower cost compared with replacing the conventional weather radar antenna array with a fully digital array supporting the phased-MIMO mode; (iii) convenient field data collection to support pioneering research in MPAR and help the authorities to decide whether it is worth it to incorporate these newly emerged antenna techniques that are widely acclaimed in the research community into real antenna design.

The rest of this work is organized as follows: In Section 2, the methodology of the proposed complementary waveform design strategy is described. In Section 3, the basic working mechanism of dual-polarized weather radar is explained. In Section 4, experimental results are presented and analyzed. Section 5 concludes this article.

## 2. Complementary Design of Two Types of Time–Frequency-Multiplexed Signals

**Figure 3:**
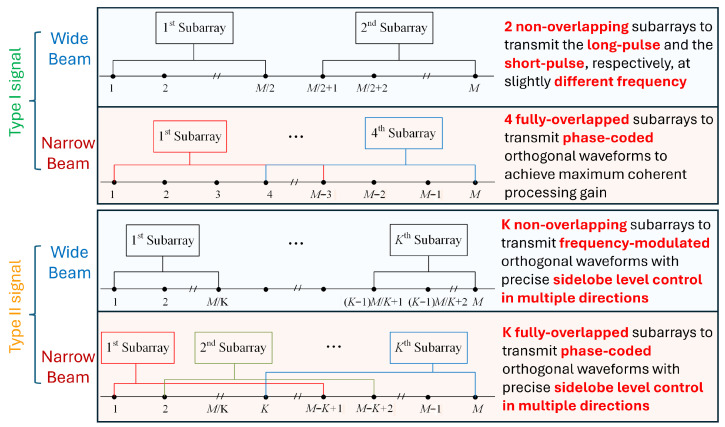
The proposed signaling strategy.

The signaling strategy is illustrated in Figure 3. If the scan update frequency is the priority and a wide beam is required, the phased-MIMO array AuxAnt 1 and 2 are divided into two non-overlapping subarrays to transmit FM waveforms in long pulses and short pulses, respectively, so that the blind zone is minimized while maintaining a balance between the maximum detection range and resolution [25,27]. To reduce range sidelobes, the induced echo signals go through the APC branch rather than conventional matched filter [18,19,20]. In the case that sensitivity and angular resolution are the priorities and a narrow main beam is required, AuxAnt 1 and 2 are partitioned into four fully overlapped subarrays to transmit PC orthogonal waveforms, with each subarray consisting of M−3 antenna elements.

In comparison, the Type II signal transmitted by AuxAnt 3 and 4 is automatically generated based on the contextual information and the principle of complementariness within the framework of cognitive radar [28]. For example, if the beams transmitted by AuxAnt 1 and 2 are pointed forward, then the beams transmitted by AuxAnt 3 and 4 are steered to a depression angle of 30° hence the terrain information is fed to AuxAnt 3 and 4 for ground clutter suppression. For the transmission of a wide main beam covering the spatial sector Θ with precisely controlled sidelobe levels in certain directions, AuxAnt 3 and 4 are divided into *K* non-overlapping subarrays transmitting FM signals that are separated in frequency, so that the signals could be separated at the receiver to exploit the waveform diversity gain. For the transmission of a narrow main beam pointing at θ with predetermined sidelobe levels in multiple directions during one radar pulse, AuxAnt 3 and 4 are divided into *K* fully overlapped subarrays transmitting PC signals to exploit the coherent processing gain.

Pulse repetition frequency (PRF) selection should be decided according to the expected velocity precision and the maximum range. The Nyquist velocity is given by PRFλ/4 and the maximum unambiguous range is c/(2PRF). For an airborne weather radar, the maximum range should be at least greater than the distance from the aircraft to the ground surface if the weather target is the updrafts of a thunderstorm. For example, if the PRF is 4400 Hz, it results in a Nyquist velocity of 34.4 m/s and a maximum range of 34.1 km, which is enough for most aircrafts. In contrast, if longer slant ranges are required, the PRF has to be lowered, leading to a reduced Nyquist velocity. If a simple pulse train is employed, pulse widths from 0.25 to 1 μs lead to range sampling intervals from 37.5 m to 150 m. For most of precipitation measurements, it is required that the vertical resolution be finer than 75 m. Doubling the pulse width could improve the sensitivity by 6 dB, but the range resolution would be worsen to twice the original value. To counter this problem, pulse compression based on frequency modulation and phase-coding is employed for wide and narrow main beams, respectively, which is detailed in Section 2.1.

### 2.1. Type I Signal Design and Receive Processing

The waveform selection is based on the trade-off between sidelobe level, mainlobe width, Doppler tolerance, and bandwidth limitations. The peak sidelobe level (PSL) for a 13-bit Barker-coded waveform is −22.3 dB, while the PSL for LFM could be reduced to −52 dB. Moreover, to use Barker phase-coded waveforms, the likely Doppler mismatch has to be limited to a one-quarter cycle or less across the pulse, i.e.,

(1) $$\begin{array}{c} F_{D_{max}}\tau <\frac{1}{4},\;v_{max}<\frac{\lambda_{t}}{8\tau }. \end{array}$$

For a radar pulse width of 67 μs, the Doppler mismatch is limited to 3.73 kHz. If the radar is X-band (10 GHz), the maximum velocity mismatch becomes 60 m/s. Beyond this critical value, the matched filter output becomes distorted, with the peak amplitude reduced, range resolution degraded, and sidelobe levels increased. In contrast, the only noticeable side effect brought by a Doppler shift of 50 m/s for the PX1000 radar developed by University of Oklahoma, Norman, Oklahoma, OK, USA between 2008 and 2012 [24] that employs the frequency-modulated waveform is an increased peak sidelobe level of −40 dB on one side of the mainlobe, while the peak amplitude, the mainlobe width, and integrated sidelobes remain constant [29]. This means that a frequency-modulated (FM) waveform is preferable for convective cells that not only grow continuously in the elevation dimension but also involve heavy rain and precipitation attenuation. If the goal is to estimate the meteorological variables for the top of a storm cell which contains ice crystals, then a phase-coded (PC) waveform could be used. Thanks to the transmit beampattern design flexibility provided by the phased-MIMO antenna configuration, the PC waveform is transmitted by the narrow main beam if sensitivity and angular resolution are the priority, while the FM waveform is transmitted by the wide main beam if the scan update times are the priority.

#### 2.1.1. FM Waveform Design for Wide Main Beam

The phased-MIMO array AuxAnt 1 and 2 are divided into two non-overlapping subarrays to transmit the long pulse and the short pulse, respectively, which is equivalent to the twin transmit–receive channel architecture employed by PX1000. The two subarrays transmit at slightly different frequencies, so that the echoes associated with the long pulse and the short pulse could be separated at the receiver end for FAPC processing. Based on the weather radar equation, the reflectivity factor is given by [25]

(2) $$\begin{array}{c} Z=\frac{R^{2}L^{2}{\left(4\pi \right)}^{3}16\ln2P_{r}\lambda^{2}}{P_{t}G^{2}c\tau \pi^{6}\theta^{2}{\left|k_{w}\right|}^{2}}, \end{array}$$

where $P_{t}$ and $P_{r}$ are the power of the transmitted and the received signal, respectively (*W*); *G* is the gain for the antenna; *L* is the loss; *c* is the speed of light (ms^−1^); τ is the pulse width (s); λ is the radar wavelength (m); *R* is range (m); θ is the one-way half-power beamwidth (rad); and $k_{w}$ is the dielectric factor of water that is decided by the temperature.

#### 2.1.2. PC Waveform Design for Narrow Main Beam

To maximize the coherent processing gain, AuxAnt 1 and 2 are partitioned into four fully overlapped subarrays, with each subarray consisting of M−3 antenna elements. The transmit steering vector of the *k*-th subarray (k=1,…,4) in the direction θ is given by

(3) $$\begin{array}{c} a_{k|FS}\left(\theta \right)=p_{k|FS}\circ a\left(\theta \right) \end{array}$$

where ∘ represents the Hadamard product; $p_{k|FS}$ is given by

(4) $$\begin{array}{c} p_{k|FS}\left(m\right)=\left\{\begin{array}{c} 0,\;m=1,2,\cdots ,k-1,\\ 1,\;m=k,k+1,\cdots ,k+M-4,\\ 0,\;m=k+M-3,\cdots ,M, \end{array}\right. \end{array}$$

so that M−3 antenna elements are active for the *k*-th subarray and a is the M×1 steering vector given by

(5) $$\begin{array}{c} a\left(\theta \right)={\left[1\;e^{-j2\pi \frac{d\sin\theta }{\lambda }}\cdots \;e^{-j2\pi \left(M-1\right)\frac{d\sin\theta }{\lambda }}\right]}^{T}. \end{array}$$

where λ is the radar wavelength.

#### 2.1.3. FAPC

The APC based on the RMMSE was first proposed by Blunt and Gerlach in 2003–2004 [30,31], and was tested with numerous simulations in 2005–2007 [19,32,33]. Taking into account the difficulty involved in replacing the matched filter (MF) in the existing radar system with the adaptive filter, Blunt and Gerlach proposed the pulse compression repair (PCR) algorithm [20]. The PCR has the same mathematical expression as the RMMSE estimation algorithm, but it works on the MF output to deal with the masking effects of the range sidelobes. The cost function of the APC filter is given by

(6) $$\begin{array}{c} J\left(l\right)=E[|x\left(l\right)-w^{H}\left(l\right)y\left(l\right){|}^{2}] \end{array}$$

where *l* represents the range cell number, w is the weighting vector, x(l) is the *l*-th sample of the range profile impulse response, and y(l) is the received signal. Assuming that the length of the sampled version of the transmitted waveform is *N*, then y(l) is given by

(7) $$\begin{array}{c} y\left(l\right)={\left[y(l)\;y(l+1)\cdots y(l+N-1)\right]}^{T} \end{array}$$

**Figure 4:**
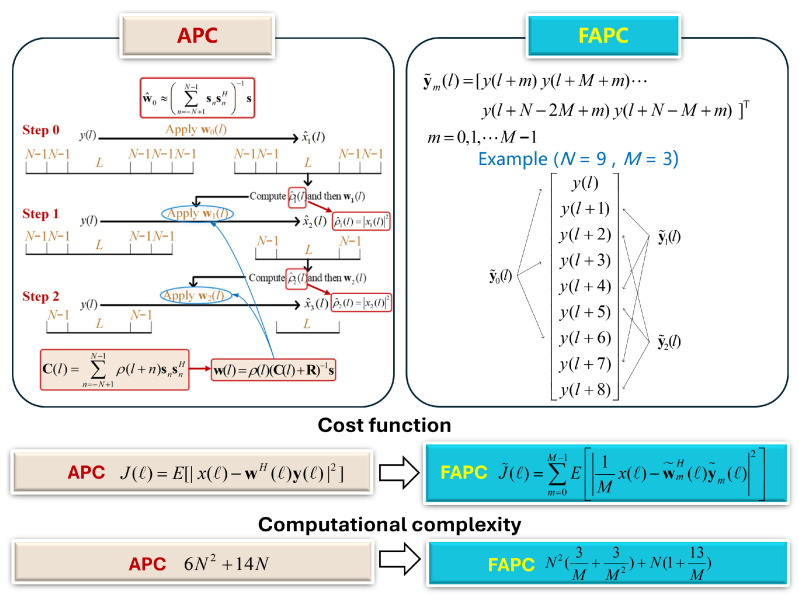
Comparison between the APC and the FAPC technique.

The flowchart of the APC technique is shown in Figure 4, where s and R represent the transmitted waveform and the interference covariance accounting for clutter. $s_{n}$ represents shifting the elements in the vector by *n* positions. For example, $s_{2}={\left[0\;0\;s_{0}\cdots s_{N-3}\right]}^{T}$, $s_{-2}={\left[s_{2}\cdots s_{N-1}\;0\;0\right]}^{T}$. Although the APC technique produces much lower range sidelobes than the conventional matched filters (MFs), its computational complexity is very high and is not suitable for implementation in real-time weather radar signal processing. Thereby, Blunt and Higgins later proposed the fast APC (FAPC) [34,35] to reduce the computational load at the cost of moderate performance degradation. The essence of the FAPC algorithm is to divide the received signal vector y(l) into *M* segments, and the cost function of the FAPC filter is given by

(8) $$\begin{array}{c} \tilde{J}\left(l\right)=\sum_{m=0}^{M-1}E\left[{\left|\frac{1}{M}x\left(l\right)-{\tilde{w}}_{m}^{H}\left(l\right){\tilde{y}}_{m}\left(l\right)\right|}^{2}\right] \end{array}$$

where ${\tilde{y}}_{m}\left(\ell \right)$ is the *m*-th segment.

### 2.2. Context-Driven Type II Signal Generation and Receive Processing

To ensure the safety of the aircraft in the landing process, it would be beneficial to capture the full picture of a growing storm cell in the elevation dimension. It follows that if the beams transmitted by AuxAnt 1 and 2 are pointed forward, then the beams transmitted by AuxAnt 3 and 4 should be steered to a depression angle pointing downwards. Consequently, the Type II signal transmitted by AntAux 3 and 4 should be adaptively generated based on the internal terrain database and the field data collected by AuxAnt 1 and 2 for clutter suppression. Thanks to the phased-MIMO antenna structure, AuxAnt 3 and 4 with *K* subarrays could transmit *K* orthogonal waveforms with precise sidelobe level control in multiple directions during one radar pulse. The echo signals associated with different transmit waveforms could be separated at the receiver end for coherent processing at the subarray level.

#### 2.2.1. Wide Main Beam Based on FM

The FM waveform is transmitted by the wide main beam if the scan update frequency is the priority. At present, to achieve higher target Doppler estimation accuracy and better clutter suppression performance, the aperture switching (AS) strategy based on multichannel receiving and digital beamforming is often employed. Specifically, on transmit, the whole radar antenna array forms a full transmit aperture; on receive, the radar antenna array is partitioned into two subarrays halfway to form two receive channels, and the signals received by the two subarrays separately are combined for every second pulse. This switching strategy has been proven to be effective in airborne radar target velocity estimation and clutter suppression in many works with real field data [36,37,38]. In this work, leveraging the flexibility provided by the phased-MIMO structure, we expand the conventional single-TX multi-RX configuration to a multichannel TX-RX configuration by dividing AuxAnt 3 and 4 into *K* non-overlapping subarrays. The transmit steering vector of the *k*-th subarray is given by

(9) $$\begin{array}{c} a_{k|NS}\left(\theta \right)=p_{k|NS}{}^{\circ}a\left(\theta \right) \end{array}$$

where

(10) $$\begin{array}{c} p_{k|NS}\left(m\right)=\left\{\begin{array}{c} 0,\;m=1,2,\cdots ,(k-1)M/K,\\ 1,\;m=(k-1)M/K+1,\cdots ,kM/K,\\ 0,\;m=kM/K+1,\cdots ,M, \end{array}\right. \end{array}$$

The transmit–receive steering vector is thus given by

(11) $$\begin{array}{c} s_{NS}\left(\theta \right)=d\left(f_{d}\right)⊗\left\{\left[c_{NS}\left(\theta \right)\right]⊗b\left(\theta \right)\right\} \end{array}$$

where

(12) $$\begin{array}{c} c_{NS}\left(\theta \right)={\left[w_{1|NS}^{H}a_{1|NS}\left(\theta \right)e^{-j\tau_{1|NS}\left(\theta \right)},\cdots ,w_{K|NS}^{H}a_{K|NS}\left(\theta \right)e^{-j\tau_{K|NS}\left(\theta \right)}\right]}^{T} \end{array}$$

$\tau_{k|NS}\left(\theta \right)$ in (12) is given by

(13) $$\begin{array}{c} \tau_{k|NS}\left(\theta \right)=2\pi k\left(M/K\right)\left(d/\lambda \right)\sin\theta \sin\varphi \end{array}$$

Suppose that the spatial sector of interest is Θ and the sidelobe region is $\bar{\Theta }$, then the weighting vector in (12) is decided by solving the following optimization problem

(14) $$\begin{array}{cc} \min_{w_{k}} & \max_{\theta }\;\left|\left|G_{d}\left(\theta \right)\right|-\left|w_{k}^{H}a_{k|NS}\left(\theta \right)\right|\right|,\theta \in \Theta , \end{array}$$

(15) $$\begin{array}{cc} s.t.\; & w_{k}^{H}a_{k|NS}\left(\theta \right)\leq \epsilon ,\theta \in \bar{\Theta } \end{array}$$

(16) $$\begin{array}{cc} \; & w_{k}^{H}a_{k|NS}\left(\theta_{j}\right)=\Delta_{k},j=1,2,\cdots ,J, \end{array}$$

where $G_{d}\left(\theta \right)$ is the desired transmit beampattern and ϵ is for sidelobe control.

#### 2.2.2. Narrow Main Beam Based on PC

If sensitivity and angular resolution are the priority, a narrow main beam is employed. The proposed signaling strategy enables data collection with multiple beams having different sidelobes facing towards multiple directions, which lays the foundation for selective data fusion based on the data collected by AuxAnt 1 and 2 later and the development of the storm cell. Assume that the main beam direction is $\theta_{0}$ and the highest allowable sidelobe level towards direction $\theta_{j}$ is $\eta_{max}$. If $\Psi_{1}$ is taken as the reference waveform and $w_{1}$ is the mother transmit beamforming weight vector, it follows that the transmit gains towards $\theta_{j}$ associated with the mother beamforming vector and the other beamforming vectors are, respectively, given by

(17) $$\begin{array}{cc} G_{1} & =w_{1}^{H}a_{1|FS}\left(\theta_{j}\right), \end{array}$$

(18) $$\begin{array}{cc} G_{k} & =w_{k}^{H}a_{k|FS}\left(\theta_{j}\right),k=2,\cdots ,K. \end{array}$$

The mother transmit beamforming weight vector $w_{1}$ is determined by solving the following optimization problem

(19) $$\begin{array}{cc} \min_{w_{1}}\; & \max_{\theta }\;\left|w_{1}^{H}a_{1|FS}\left(\theta \right)\right|,\theta \in \left[0,\theta_{0}-b_{hw}\right]\cup \left[\theta_{0}+b_{hw},\pi \right], \end{array}$$

(20) $$\begin{array}{cc} s.t.\; & w_{1}^{H}a_{1|FS}\left(\theta_{0}\right)=1 \end{array}$$

(21) $$\begin{array}{cc} \; & w_{1}^{H}a_{1|FS}\left(\theta_{j}\right)=\eta_{\max},j=1,\;2,\cdots ,J. \end{array}$$

where $\theta_{0}$ is the main beam direction and $b_{hw}$ is the half-beam width.

## 3. Dual-Pol Weather Radar Basics

Dual-polarization (dual-pol) weather radar provides better hydrometeor classification performance and more accurate quantitative precipitation estimation than its single-pol counterpart. Dual-pol radar could generally be implemented in three modes: the alternate transmission and alternate reception (ATAR) mode, the alternate transmission and simultaneous reception (ATSR) mode, and the simultaneous transmission and simultaneous reception (STSR) mode. Among these three modes, the STSR mode, where the transmitted power is split into two channels (H and V) for transmission and the echo signals are received in two channels (H and V) simultaneously, is the most popular approach and is supported by the proposed auxiliary antenna arrays.

First, the polarimetric radar data could contribute to more accurate hydrometeor classification. For example, the reflectivity induced by heavy rain is quite similar to that induced by light hailstones with water shells, which makes them undistinguishable for single-pol radar. In contrast, dual-pol weather radar could easily distinguish the two by exploiting the differential reflectivity, $Z_{DR}$, which conveys information regarding the shape of the particles and the dielectric constant, and the correlation coefficient, $\rho_{hv}$. Generally speaking, $Z_{DR}$ increases with the size of the raindrops since larger raindrops are more oblate. Moreover, raindrops have much higher ZDR compared with snowflakes/graupel of a similar size, which makes $Z_{DR}$ critical for discrimination between liquid and frozen hydrometeors. Meanwhile, $\rho_{hv}$ measures the diversity (i.e., types/shapes/orientations) of particles within a radar sampling volume, and has a value close to 1 for pure rain and snow.

Second, the polarimetric radar data are critical for rainfall estimation. The total differential phase measured by the polarimetric radar, $\Psi_{DP}\left(r\right)$, consists of two parts, the differential scattering phase δ(r) and the differential (propagation) phase $\phi_{DP}\left(r\right)$. The derivative of $\phi_{DP}\left(r\right)$ with respect to *r* is termed as the specific differential phase and is commonly represented by $K_{DP}$. $K_{DP}$ is inversely propositional to the radar wavelength and is approximately proportional to the concentration of particles. It has been shown in the latest research that for reflectivity greater than 45 dBz and $K_{DP}$ greater than 0.1°km^−1^, the rainfall rate *R* (in mm h^−1^) could be more accurately estimated based on $K_{DP}$ [39]. As a result, the quantitative precipitation estimation (QPE) is improved from 35% root mean square errors (RMSEs) to about 10–15% RMSEs by introducing dual polarization [40].

**Figure 5:**
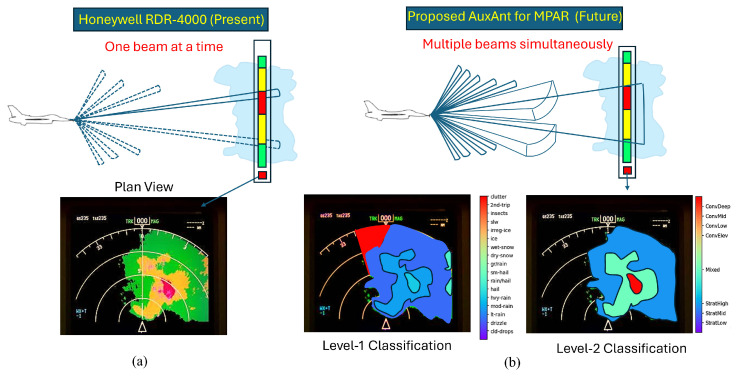
The advantage of the proposed AuxAnt for MPAR over the conventional weather radar. (**a**) Honeywell RDR-4000. Four colors (green, yellow, red, magenta) are used to represent four radar signal levels; weather classification is not available. (**b**) Avionic radar with the proposed AuxAnt and signaling strategy, which enables accurate weather classification.

Since the major purpose of this work is to demonstrate the advantages that could be brought about by the proposed antenna architecture and signaling strategy rather than developing an avionic weather radar simulator, we leverage the 3D weather radar data collected by the ground-based dual-pol radars as the ground truths. Normally, the cruise altitudes for propeller-drive aircrafts, subsonic airliners, and supersonic transport are 2000–15,000 feet (610–4572 m), 20,000–40,000 feet (6096–12,192 m), and 60,000–70,000 feet (18,288–21,336 m), respectively, while the full 3D grid of the reflectivity field provided by the ground-based weather radars covers up to 20 km in the elevation dimension. This means that the vertical distribution of the weather during a typical weather phenomenon, e.g., convective or stratiform precipitation, recorded by multiple widely distributed ground-based weather radars could be utilized for simulating avionic weather radar echoes received from different altitudes. Specifically, the reflectivity map displayed on the screen of the avionic radar (e.g., RDR4000) is a composite image collected over many sweeps with various antenna tilt angles. If the maximum reflectivity for the region of interest at any altitude passes the threshold for “heavy precipitation”, then that region is marked as red, thereby reducing the overall risk for the aircraft to pass through a growing storm cell, which often features a less reflective storm cell top and a highly reflective storm center. This process is illustrated in Figure 5a. By employing a dual-pol antenna and transmitting multiple narrow/wide beams simultaneously, an avionic radar with the proposed AuxAnt and signaling strategy could provide more detailed information about a potentially dangerous weather phenomenon for a larger region in a more timely manner, which is illustrated in Figure 5b. The output of this module is a .mat file featuring 3D grids, where each point of the volume is described by the temperature, pressure, and statistics of hydrometeors. The difference between X-band and S-band radars could be accounted for based on the results presented by Barcaroli et al. [41].

## 4. Experimental Results

### 4.1. Performance Improvement Brought About by Dual-Pol Configuration and Multibeam Scan

**Figure 6:**
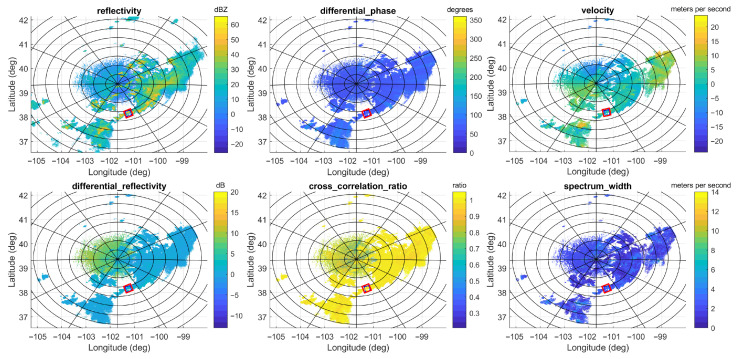
The primary variables of the dual-pol radar data collected by the WSR-88D dual-pol weather radar during precipitation occurring at 22:00 on 7 July 2021 in Goodland, Kansas, which include reflectivity, differential phase, radial velocity, differential reflectivity, cross correlation ratio, and spectrum width. The area in the red square corresponds to the ROI.

**Figure 7:**
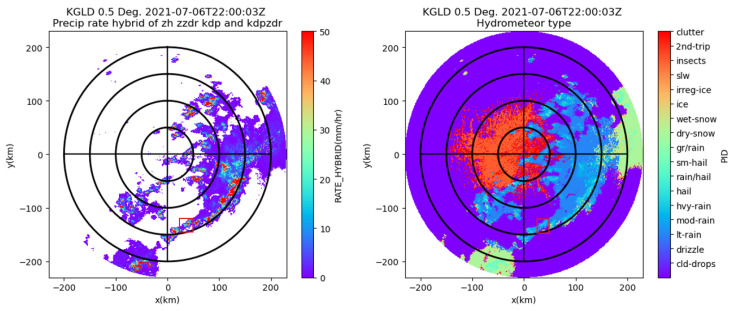
The precipitation rate estimation result (**left**) and the hydrometeor type prediction result based on the data collected by the WSR-88D radar at the KGLD site (**right**).

Due to the lack of avionic weather radar data in the public domain, the data recorded by the ground-based WSR-88D dual-pol weather radar are used as the ground truth in this subsection. Specifically, we determine that the precipitation occurred at 22:00 on 7 July 2021 in Goodland, Kansas. The primary variables of the dual-pol radar data collected by the WSR-88D dual-pol weather radar at the KGLD site are illustrated in Figure 6, which include reflectivity, radial velocity, differential reflectivity, differential phase, and specific differential phase. The region of interest (ROI) is between 38.0° N and 38.3° N in latitude and −101.4° and −101.1° in longitude and is marked with a red square. The center of the ROI is 130 km south, 40 km east of the KGLD radar. The precipitation rate estimation result and the hydrometeor type prediction result based on the WSR-88D radar data are provided in Figure 7, where the area in the red square corresponds to the ROI.

**Figure 8:**
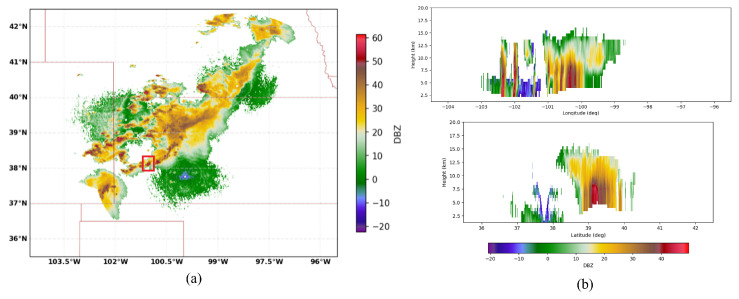
The 3D reflectivity data reconstructed by jointly exploiting the contributions made by three WSR-88D radars and the vertical sections. (**a**) The 3D reflectivity data. (**b**) Longitude/latitude cuts. The area in the red square corresponds to the ROI.

**Figure 9:**
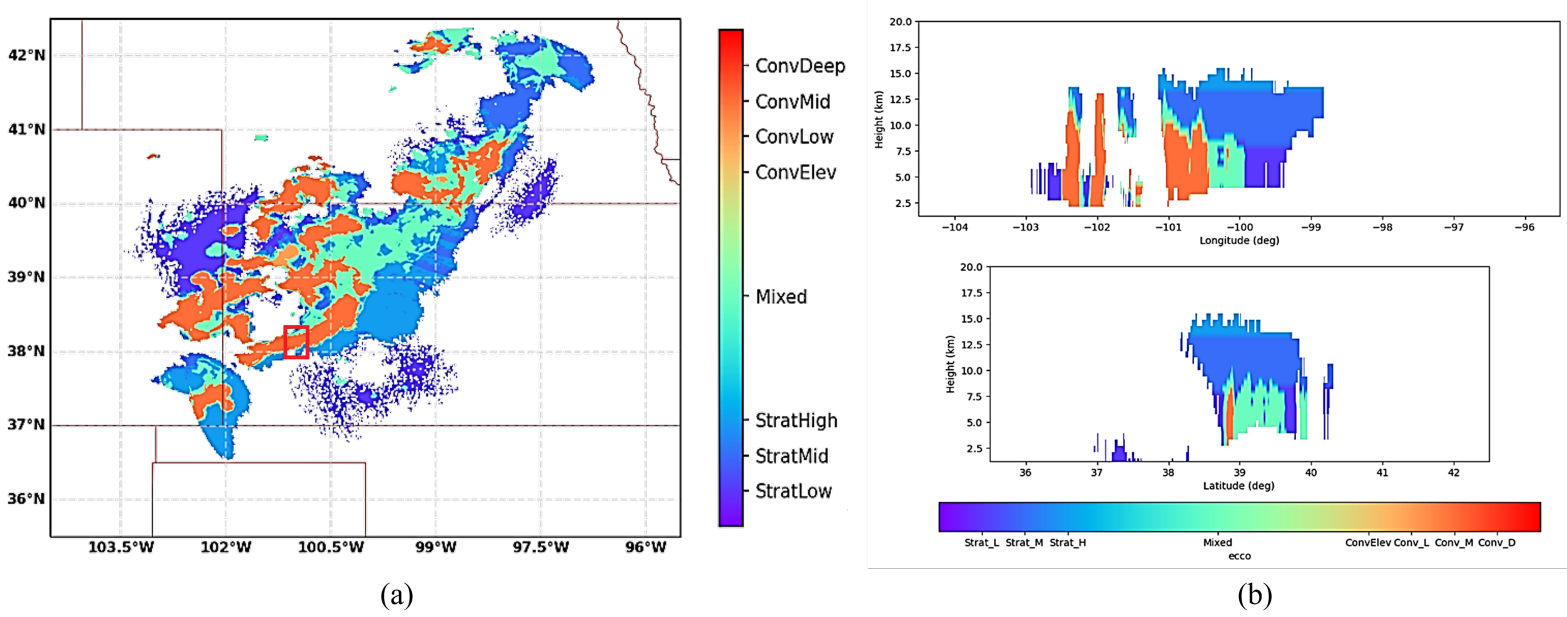
Precipitation classification results obtained with the ECCO algorithm. (**a**) Horizontal plane. (**b**) W-E and N-S vertical sections corresponding to the location of the maximum dBz value.

The column-max reflectivity data reconstructed by jointly exploiting the contributions made by three WSR-88D radars (the KGLD site in Goodland, Kansas; the KUEX site in Hastings, Nebraska; and the KDDC site in Dodge, Kansas) are illustrated in Figure 8a. Figure 8b illustrates the West–East (W–E) vertical section and the North–South (N–S) vertical section, respectively, corresponding to the location of the maximum dBz value. The geographical coordinates of the KUEX, KDDC, and KGLD radars are ($40.320833^{{}^{\circ}}$ N, 98.441944° W), (37.760833° N, 99.968889° W), and (39.366944° N, 101.70027° W), respectively. The elevations of the KUEX, KDDC, and KGLD radars are 2057, 2671, and 3716 feet, respectively. The vertical axis has 31 levels from 1.5 km to 19 km in altitude, with 0.25 km spacing up to 3 km, 0.5 km vertical spacing from 3 to 9 km, and 1 km spacing from 9 to 19 km. Based on these data, precipitation is further classified as convective, mixed, and stratiform based on the Echo Classification from COnvectivity (ECCO) algorithm [42]. It can be seen from Figure 7, Figure 8 and Figure 9 that the ROI is experiencing a ConvDeep precipitation with mixed graupel, small hail, and rain, and the storm cell in this region varies dramatically with height.

**Figure 10:**
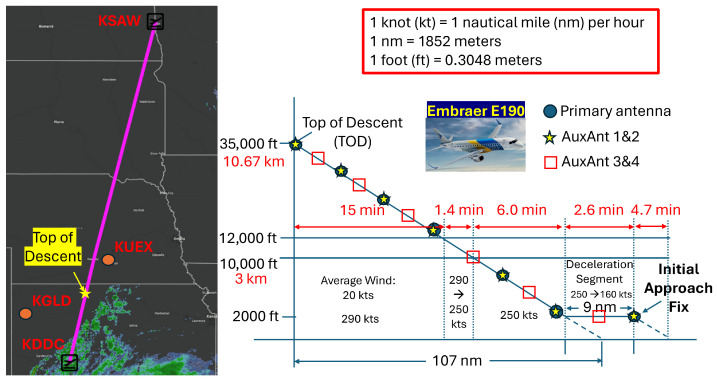
The descent profile of a commercial aircraft from the Hector International Airport (code: KSAW) in Fargo, North Dakota, to Dodge City Regional Airport (code: KDDC), Dodge City, Kansas.

To demonstrate the advantage of the performance improvement brought by the dual-pol configuration, we consider the standard aircraft descent scenario illustrated in Figure 10, where a commercial aircraft is flying from the Hector International Airport (code: KSAW) in Fargo, North Dakota, to Dodge City Regional Airport (code: KDDC), Dodge City, Kansas. The total time duration of the descent is 30 min; the top of the descent is 116 nautical miles (215 km) from the destination and is marked with a star symbol on the left of Figure 10. The moving speed and altitude of the aircraft at different stages are marked on the right of Figure 10. To illustrate the working mechanism of the proposed auxiliary antennas, the antenna sweep schedules for the primary antenna, AuxAnt1 and 2, as well as AuxAnt 3 and 4 are depicted with blue circles, yellow stars, and red squares.

**Figure 11:**
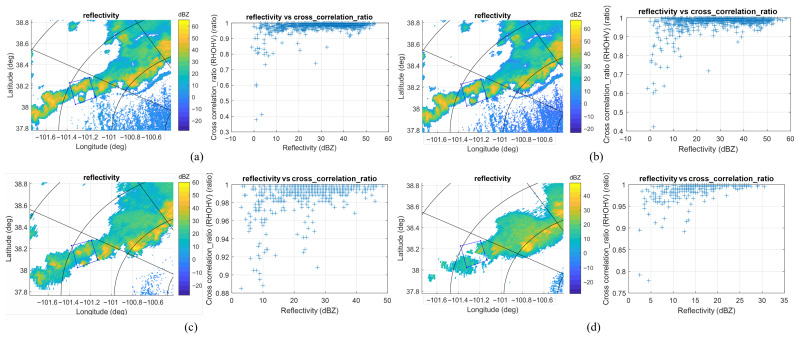
The reflectivity and cross correlation ratio for the ROI acquired by KDDC with two different tilt angles. (**a**) KDDC at 22:00:00, tilt = 0.5°. (**b**) KDDC at 22:02:09, tilt = 0.5° (MRLE re-scan). (**c**) KDDC at 22:02:48, tilt = 2.4°. (**d**) KDDC at 22:03:14, tilt = 4°.

**Figure 12:**
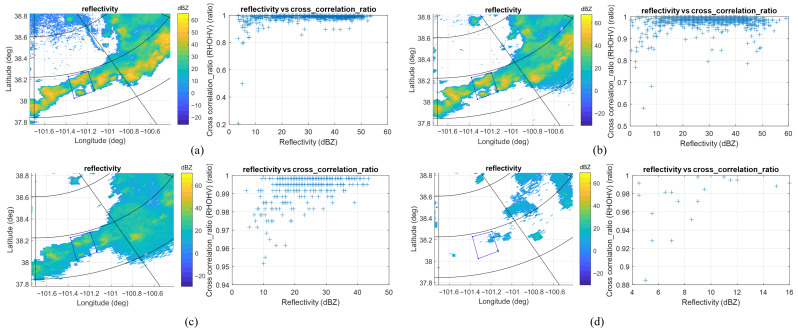
The reflectivity and cross correlation ratio for the ROI acquired by KGLD with different tilt angles. (**a**) KGLD at 22:00:03, tilt = 0.5°. (**b**) KGLD at 22:01:28, tilt = 1.3°. (**c**) KGLD at 22:02:23, tilt = 2.4°. (**d**) KGLD at 22:02:49, tilt = 4°.

The reflectivity and cross correlation ratio for the same region acquired by KDDC and KGLD with different tilt angles are illustrated in Figure 11 and Figure 12, respectively. The ROI is 115 km north, 45 km west of the KDDC radar. For the KDDC radar, the precipitation echo signals fall below the detection threshold for a tilt angle greater than 5°; for the KGLD radar, the precipitation echo signals become undetectable when the tilt angle is equal to or greater than 4°, which demonstrates the necessity of simultaneous multibeam scanning with the proposed antenna configuration.

To quantify the performance improvement brought about by multibeam scan, the flight path weather data collected by the KDDC radar (red text), KGLD radar (green text), and KUEX radar (blue text) are summarized in Table 1. The data in the yellow-, purple-, and grey-shaded cells are used for weather estimation for the scenario depicted in Figure 10 at three time checkpoints (22:07:23 ± 60 s, 22:12:04 ± 60 s, 22:16:00 ± 60 s), respectively. The locations of interest correspond to these time checkpoints, i.e., the future time of interest, and when and where the weather observations are made are summarized in Table 2, where “Lat.” and “Long.” stand for latitude and longitude, respectively. To simulate the worst scenario, the storm cell is moved 0.5° south and 0.5° east to a position where it could have serious impact on the flight if undetected. The advantage of the proposed dual-pol auxiliary phased-MIMO antenna arrays with the multibeam scanning strategy over the conventional single-pol avionic weather radar is shown in Table 3, where “Reflect.”, “Meas.”, and “pred.” stand for reflectivity, measurement, and prediction, respectively. Since the reflectivity map displayed on the screen of the avionic radar is a composite image collected over many sweeps with various antenna tilt angles, the maximum reflectivity of the weather data collected by multiple beams is recorded as the measurement made by the proposed antenna. In addition, due to the joint employment of the dual-pol mode and the Type I/II signal, the precipitate can be classified as ConvDeep, ConvMid, ConvLow, ConvElev, Mixed, StratHigh, StratMid, and StraLow based on the ECCO algorithm, similarly to the case shown in Figure 9, while the conventional single-pol weather radar can only classify the precipitation according to intensity. Finally, by employing the auxiliary phased-MIMO antenna arrays, the data rate is increased while the time that is required to scan ±4000 feet around the flight track is reduced by multiple times (the specific performance metric depends on *K*, the number of subarrays, the beamwidth, and the relative position between the radar and the future time and location of interest).

### 4.2. Performance Improvement Brought About by Time–Frequency Multiplexing

**Figure 13:**
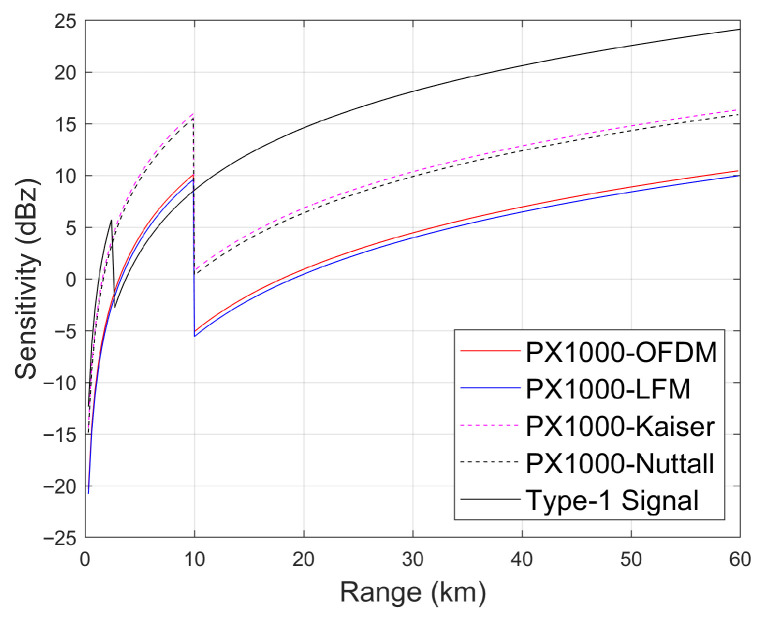
Comparison between the sensitivities of the radars presented in Table 4.

The key parameters of the WXR-2100 MultiScan^TM^, the IntuVue^®^(RDR-4000), the PX1000, and the proposed X-band airborne weather radar are compared in Table 4. WXR-2100 MultiScan^TM^ was developed by Rockwell Collins Inc., headquartered in Cedar Rapids, IA, USA (now part of the Collins Aerospace Inc. after the 2018 merger), in 2013 [15]. IntuVue^®^(RDR-4000) was manufactured by Honeywell Aerospace Inc., Phoenix, AZ, USA in 2012 [1]. PX1000 is a solid-state software-defined weather radar developed by Advanced Radar Research Center, University of Oklahoma, Norman, OK, USA between 2008 and 2012 [24]. It has twin independent transmit-receive chains (i.e. independent up-down converters and power amplifiers) so that each channel can transit different waveforms. The sensitivities of these radars are compared in Figure 13. Note that the abrupt sensitivity changes at 1.8 km and 10 km correspond to the blind range for the proposed radar and the PX1000 operating with interlaced long–short pulses, respectively. Based on the theoretical sensitivity comparison results presented by Kurdzo et al. [25], for the PX-1000 radar, which has a noise floor of −110 dBm, an abrupt change in sensitivity is observed at 10 km, which corresponds to the blind range for the long pulse, due to the employment of a short (fill) pulse. Since the short pulse has a pulse width of 2 μs and a bandwidth of 2.8 MHz and the long pulse has a pulse width of 67 μs and a bandwidth of 2.2 MHz, the sensitivity values provided by these two types of pulses are 15 dBZ and 0 dBz, respectively, under the assumption of a non-windowed LFM waveform.

Note that the PX-1000 radar is a ground-based radar with an antenna dish with a diameter of 1.2 m and a nominal gain of 38.5 dBi as well as a transmit power of 100 W. In contrast, it is more difficult to obtain accurate reflectivity and Doppler measurements due to SWap constraints as well as the negative effects brought about by the moving-platform effects, the natural/manmade interferences, and the air turbulence. Here we consider threerepresentative airborne weather radars for large and small aircrafts: the IntuVue RDR-4000 and the IntuVue RDR-7000 developed by Honeywell. The IntuVue RDR-4000 has a transmit power of 40 watts and employs a pulse compression ratio of 23, which results in an effective transmitter power of 917 W and an effective pulse width of 12 μs. The flat plate antenna for the IntuVue RDR-7000 developed by Honeywell for small business aircrafts (e.g., Falcon 900) and helicopters (e.g., Airbus H135) comes with two size configurations, 30 cm and 46 cm (diameter), and the transmit power is only 70 W. As a result, although the theoretical simulations and the field measurements obtained by ground-based weather radar converge well [43], it has been demonstrated in the Italian Avionic X-band Weather signal modeling and processing vALidation (X-Wald) project that the simulated reflectivity for airborne weather radars could be 10 dBz lower than the measured data [44]. According to the relationship between the rainfall rate and the reflectivity for a typical X-band airborne weather radar summarized in Table 5, strong rain could be mistaken for medium rain while light rain could be missed altogether.

### 4.3. Performance Improvement Brought About by Pulse Compression and FAPC

**Figure 14:**
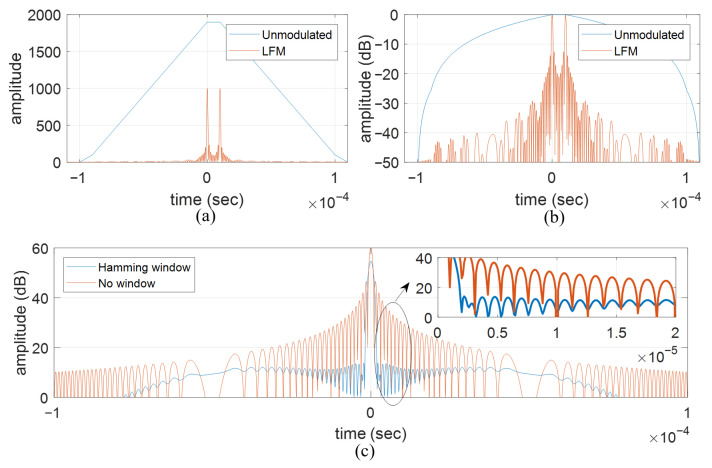
The range sidelobe suppression performance of the APC and the FAPC. (**a**) Matched filter output in numerical value. (**b**) Matched filter output in dB. (**c**) Sidelobe suppression with Hamming window.

Pulse compression is a widely adopted technique to resolve the dilemma between range resolution and the effective transmit power, which are proportional and inversely proportional to the pulse width, respectively, in the case where an unmodulated rectangular pulse train is used. Figure 14a,b illustrate the advantage of the LFM waveform over the unmodulated pulse train in discerning two closely located targets. The time–bandwidth product used for simulation is 100, with pulse width and bandwidth set as 100 μs and 1 MHz, respectively. Since the two targets are 10 μs apart (1/10 of the pulse width) in the time delay for radar echoes, they are mistaken as a single target given that the unmodulated pulse train is used, but are separated when the LFM waveform is used. Since the peak-to-sidelobe-level ratio (PSLR) of the vanilla LFM waveform is far from satisfactory, extra sidelobe suppression is often required. Figure 14c depicts the sidelobe suppression performance by applying the Hamming window in the time domain.

**Figure 15:**
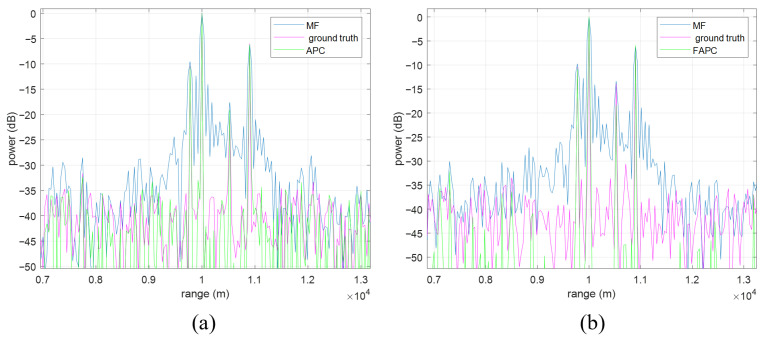
The range sidelobe suppression performance of the APC and the FAPC. (**a**) APC. (**b**) FAPC.

The range sidelobe suppression performances of the APC algorithm and the FAPC are depicted in Figure 15a and Figure 15b, respectively, where four point targets are simulated. It can be seen that, if only the MF is implemented, strong range sidelobes could be easily mistaken as targets, while the weak targets are buried in the range sidelobes of the adjacent stronger targets. Once the APC or the FAPC algorithm is implemented, the range sidelobes are efficiently suppressed, and the previously buried weak target is revealed.

### 4.4. Performance Improvement Brought About by Sidelobe Control

In the Netherlands, a dual-pol X-band radar developed by TU Delft’s International Research Centre for Telecommunications and Radar (IRCTR), which is named the IDRA (the abbreviation of IRCTR Drizzle RAdar), was installed on top of the Cabauw Tower in 2007. The IDRA dataset, which is free to access on-line, contains both the I/Q signals received by the radar and the Level II radar data in NetCDF format. During the X-Wald project, a data acquisition campaign was conducted on 16 December 2015 in the Netherlands. The radar was installed within the Diamond DA-42 pod, which took off from the Teuge airport and flew towards the Cabauw site 20 min away. Since the permission to fly in proximity of the Cabauw site was denied by the air traffic control authorities due to low-visibility conditions, the aircraft turned around and flew back to the airport after reaching Utrecht, which is 30 km from the Cabauw site. According to the plan, the IDRA data would be used as auxiliary ground measurements for the validation and optimization of the radar signal processing and weather classification algorithms. Unfortunately, the avionic weather radar failed to collect meaningful radar data despite the weak precipitation that could be visually observed from time to time.

On 15–16 March, the avionic dual-pol radar developed during the X-Wald project was carried by Piaggio P166C for a data acquisition campaign near the ground-based dual-pol weather radar POLAR 55C in Italy. Based on the airborne measurements obtained during the Italy campaign published in the technical report, the radar sensitivity is about 25–30 dBz depending on the distance, and the measured reflectivity is generally lower than the simulated one. Specifically, comparing the data acquisition performed over the Tyrrhenian sea on 16 March 2016 at a height of 250 m and the data collected on the same day above Southern Tuscany at a height of 550 m, the impact of clutter is very obvious. Since the ground-based IDRA could capture weak precipitation in the form of both rain and snow, we believe the inferior performance of the avionic weather radar is partially due to clutter and other interferences.

**Figure 16:**
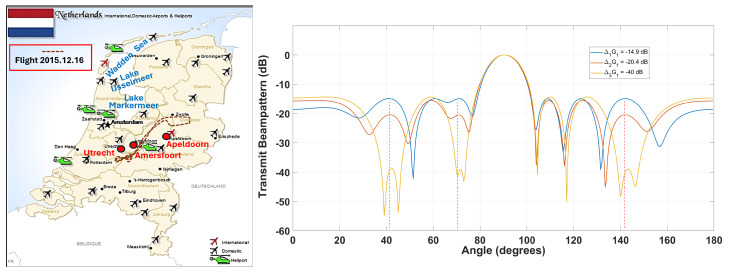
Adaptive interference mitigation over different types of terrains with sidelobe control. (**Left**): distribution of airports across the Netherlands. (**Right**): three transmit beams with predesignated SSLs in four directions formed by a phased-MIMO antenna array with 16 elements that are divided into K=3 fully overlapped subarrays during one pulse. By comparing the difference between the target echoes associated with the two waveforms, the SSL for the next pulse could be adjusted accordingly.

The aircraft trajectory during the data acquisition campaign conducted on 16 December 2015 during the X-Wald project with a flight height between 250 m and 550 m is depicted by the dash-dotted line on the left of Figure 16. It can be seen from the left side of Figure 16 that the flight trajectory chosen for the data acquisition campaign covers an area full of interferences. Specifically, helicopters often buzz above Amersfoort for law enforcement, emergency services, and infrastructure maintenance, and the Onze Lieve Vrouwetoren church tower at Amersfoort is about 100 m high. Moreover, it is clear that aircrafts in the northern part of the Netherlands need to frequently fly over water bodies (Wadden sea, Lake IJsselmeer, and Lake Markermeer), where cargo ships and small vessels come and go. Transmitting wide/narrow beams with predetermined sidelobe levels in multiple directions during each radar pulse based on contextual information provided by the on-board ground terrain database is not only beneficial for excluding heavy cluttered areas with intense inner clutter motion (ICM) and strong discrete clutter during data acquisition, but also for limiting the interferences from the weather radar to other electronic devices. The right side of Figure 16 depicts two transmit beams with predesignated sidelobe levels (SSLs) in four directions formed by a phased-MIMO antenna array consisting of 16 elements that are divided into K=3 fully overlapped subarrays (i.e., 14 elements in each subarray). By comparing the difference between the target echoes associated with the two waveforms, the SSLs for the next pulse could be adjusted accordingly.

### 4.5. Limitations and Future Works

**Figure 17:**
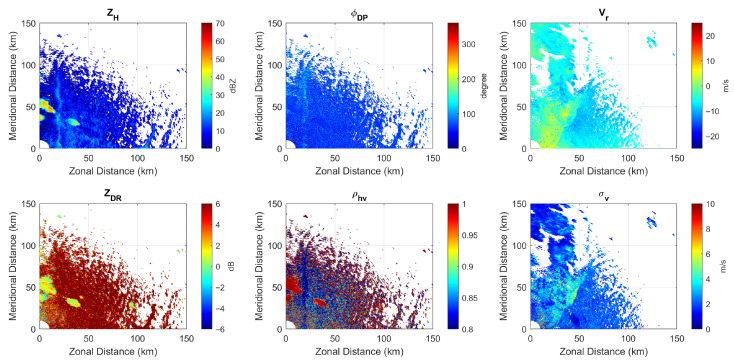
The polarimetric variables contained in the NEXRAD Level II data used in the experiment for IQ signal generation.

Since the major purpose of this work is to demonstrate the advantages that could be brought about by the proposed antenna architecture and signaling strategy rather than developing an avionic weather radar simulator, ground-based weather radar data collected by NEXRAD have been used for functional verification. In 2024, Matlab^®^ R2024a introduced a built-in function *weatherTimeSeries* that can generate time series I/Q signals for polarimetric weather radars based on the polarimetric weather variables. The radar return from a specific resolution volume is modeled as a complex stationary Gaussian random process and Monte Carlo simulation is used. To verify the fidelity of the simulated I/Q signals, the polarimetric variables contained in the data collected by the KGLD radar at 22:00:03 on 7 July 2021 with a tilt angle of 0.5° covering an area of 0–90° in azimuth and 10 km to 200 km in range are shown in Figure 17.

**Figure 18:**
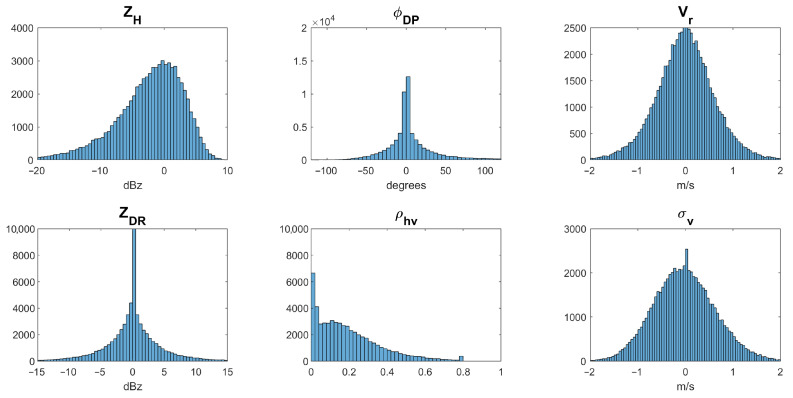
The distribution of estimation errors for six dual-pol radar variables.

**Figure 19:**
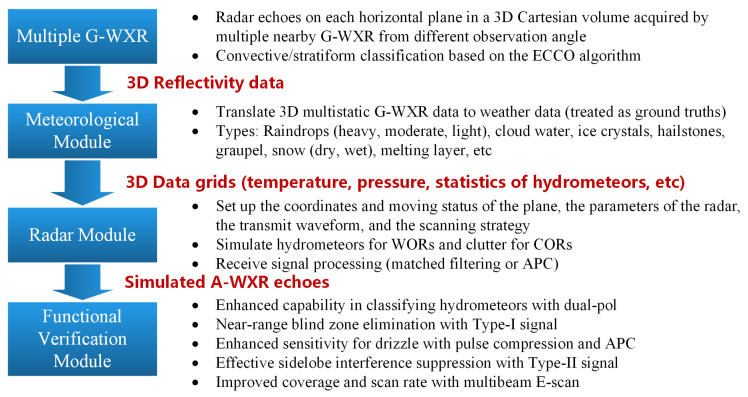
The proposed airborne weather echo simulation method for future works.

The distribution of estimation errors for six dual-pol radar variables, which are obtained by subtracting the NEXRAD ground truth from the Pulse-Pair Processing (PPP) estimates of each radar moment, is illustrated in Figure 18. It can be seen that the estimation errors are within an acceptable range, which indicates that a full simulation could be carried out in future works, as illustrated in Figure 19, where “G-WXR” and “A-WXR” represent ground-based and avionic weather radars, respectively, and “WOR” and “COR” represent weather observation regions and clutter observation regions illuminated by the radar beam, respectively.

## 5. Conclusions

Taking into account the waveform design and beamforming flexibility provided by the active electronically steered array, we propose adding four auxiliary antenna arrays based on the phased-MIMO antenna structure to the existing avionic weather radar antenna for future field data collection missions, which would be essential to support pioneering research in MPAR and help the authorities to decide whether these newly emerged antenna techniques could bring significant performance improvement in practice. Two complementary types of probing signals are designed. The Type I signal transmitted by AuxAnt 1 and 2 is designed following the classic long–short pulse interleaving principle so that the near-range blind zone is mitigated. To strike a balance between Doppler tolerance, sensitivity, resolution, and scan speed, the PC waveform is transmitted by the narrow main beam if sensitivity and angular resolution are the priority, while the FM waveform is transmitted by the wide main beam if the scan update times are the priority. To minimize the range sidelobes, FAPC based on RMMSE is employed as a substitute for the classic matched filter. The Type II signal is automatically generated based on the contextual information and the principle of complementariness. Simulation results show that by incorporating the phased-MIMO antenna technique to avionic weather radar design, the weather observation and prediction performance are enhanced dramatically.

## Figures and Tables

**Table 1:** Flight path weather data collected by the KDDC radar (red text), KGLD radar (green text), and KUEX radar (blue text). The data in the yellow-, purple-, and grey-shaded cells are used for weather estimation at 22:07:23 ± 60 s, 22:12:04 ± 60 s, and 22:16:00 ± 60 s, respectively.

Time		KDDC		KGLD		KUEX	
Sweep 0 (Start)	Sweep (tilt = 3.1°)	Distance (km)	Tilt (°)	Distance (km)	Tilt (°)	Distance (km)	Tilt (°)
**22:00:00**	**22:03:01**	**209.7**	**1.75**	201.4	1.81	114.8	4.51
**22:00:03**	**22:02:36**	209.7	1.75	**201.4**	**1.81**	114.8	4.51
**22:02:49**	**22:05:15**	186.7	1.78	197.7	1.50	**133.6**	**3.15**
**22:04:30**	**22:07:38**	**171.9**	**1.81**	194.0	1.33	147.4	2.43
**22:04:48**	**22:07:21**	169.5	1.81	**194.2**	**1.28**	149.3	2.32
**22:07:23**	**22:09:57**	146.6	1.85	191.8	0.96	**170.8**	**1.44**
**22:09:21**	**22:12:29**	**128.5**	**1.87**	191.9	0.68	188.0	0.88
**22:09:35**	**22:12:08**	126.4	1.87	**191.6**	**0.65**	190.1	0.82
**22:12:04**	**22:14:25**	104.1	1.85	192.1	0.30	**212.0**	**0.24**
**22:14:20**	**22:16:53**	84.0	1.77	**198.6**	**−0.07**	230.9	−0.21
**22:16:00**	**22:19:08**	**37.4**	**3.50**	214.8	−0.41	276.7	−0.71
**22:16:33**	**22:18:55**	36.8	3.32	214.7	−0.45	**277.3**	**−0.75**
**22:19:06**	**22:21:38**	30.2	2.00	**217.2**	**−0.76**	283.8	−1.01

**Table 2:** The locations of interest correspond to the future time of interest and when and where the weather observations are made.

Index Number	Present Time	Observation Position			Future Time of Interest	Location of Interest		
		Lat. (°)	Long. (°)	Height (m)		Lat. (°)	Long. (°)	Height (m)
1	22:00:03	39.589	−99.382	10,671	22:07:23 ± 40 s	39.032	−99.525	7219
2	22:04:30	39.254	−99.459	8567	22:12:04 ± 40 s	38.668	−99.673	5030
3	22:09:21	38.874	−99.575	6300	22:16:00 ± 40 s	38.084	−99.853	3201

**Table 3:** Performance comparison between the baseline and the proposed signaling strategy. “Mixed” stands for convective–stratiform precipitate mixture.

Index Number	Weather Ground Truth		Performance Comparison			
			Baseline		Proposed	
	Reflect. Z (dBz)	Weather	Meas. Z (dBz)	Weather Pred.	Meas. Z (dBz)	Weather Pred.
1	30	Medium + Mixed	16	Drizzle	26	Light + Mixed
2	40	Heavy + Mixed	31	Medium	41	Heavy + Mixed
3	35	Medium + Mixed	28	Light	37	Medium + Mixed

**Table 4:** Comparison between the parameters of the Rockwell Collins WXR-2100 MultiScan, the Honeywell RDR-4000, the PX1000, and the proposed X-band airborne weather radar.

Time	2013	2012	2015	2025
Radar	MultiScan version 2.0	IntuVue RDR-4000	PX1000 (2015)	Proposed Type I signal
Transmitter Power	150 watts nominal	917 watts (effective)	200 watts ×2	917 watts (effective) ×2
Radar Sensitivity	20 dBz at 9 km (5 nm)	20 dBz at 28 km (15 nm)	20 dBz at 60 km	20 dBz at 56 km (30 nm)
Transmitter Frequency	9.330 GHz	9.375 GHz	9.550 GHz	9.330 GHz
Pulse Width	Radar modes: 6 and 20 µs (interlaced) Windshear mode: 2 µs	12 µs (effective)	1–69 µs	2 and 18 µs (interlaced)
Pulse Compression	N/A	LFM	OFM	LFM
Beamwidth (Antenna Size)	3.5° (28-inch)	3° (30-inch) 4.2° (24-inch) 5.6°(18-inch)	1.8 °(47-inch)	3° (30-inch)

**Table 5:** The relationship between rainfall rate and reflectivity for a typical X-band airborne weather radar.

Color Code	Intensity of Returns	Reflectivity (dBz)	Rainfall Rate (mm/h)	Rainfall Rate (in/h)
Black	Very light	20	0.76	0.03
Green	Light	20–30	0.76–3.81	0.03–0.15
Yellow	Medium	30–40	3.81–12.7	0.15–0.5
Red	Strong	≥40	12.7	0.5
Purple	Turbulence	N/A	N/A	N/A

## Data Availability

Some of the data are available upon request.
